# Percutaneous intramedullary screw or rush pin fixation of unstable ankle fractures in patients with fragile soft tissue – retrospective study of 80 cases

**DOI:** 10.1007/s00402-024-05290-w

**Published:** 2024-04-13

**Authors:** Simon Oksbjerre Mortensen, Jeppe Barckman, Per Hviid Gundtoft

**Affiliations:** 1 Department of Orthopedic Surgery, Aarhus University Hospital, Palle Juul-Jensens Boulevard 99, Aarhus N, 8200 Denmark; 2 Department of Orthopedic Surgery Regions Hospitalet Randers, Skovlyvej 15, Randers, 8930 NØ Denmark

**Keywords:** Distal fibula fracture, Fibula rush pin, Fragile skin conditions, Re-operation, Soft tissue complications

## Abstract

**Introduction:**

The standard surgical procedure for unstable ankle fractures is fixation of the lateral malleolus with a plate and screws. This method has a high risk of complications, especially among patients with fragile skin conditions. The aim of this study was to estimate the re-operation rates and identify complications in patients with an unstable ankle fracture, surgically treated with an intramedullary screw or rush pin.

**Materials and methods:**

We identified all patients who were surgically treated with either a 3.5-mm screw or rush pin at Aarhus University Hospital, Denmark, from 2012 to 2018. Major complications were re-operations within three months. We included 80 patients, of which 55 (69%) were treated with a 3.5-mm intramedullary screw and 25 (31%) with a rush pin. The majority of the study population was female (59) and the mean age was 75 (range 24 to 100) years. Of the 80 patients included, 41 patients had more than 2 comorbidities.

**Results:**

Three patients underwent re-operation within three months due to either fracture displacement or hardware cutout. Radiographs obtained after six weeks showed that nine patients had loss of reduction. Additionally, four patients had superficial wound infections and six patients had delayed wound healing.

**Conclusions:**

Intramedullary fixation of distal fibula fractures with either a screw or rush pin has low re-operation rates. However, the high proportion of patients with radiological loss of reduction is concerning.

**Supplementary Information:**

The online version contains supplementary material available at 10.1007/s00402-024-05290-w.

## Introduction

Ankle fractures are one of the most common types of acute skeletal injuries [1]. In patients with unstable ankle fractures, the importance of accurate anatomical reduction of the lateral malleolus is well-documented [2]. The standard surgical procedure for unstable ankle fractures is open reduction and internal fixation of the lateral malleolus with a plate and screws [2]. The disadvantage of this method is that it requires extensive dissection, and with only a thin layer of soft tissue covering the lateral malleolus, this procedure is associated with a high risk of complications such as wound infections. Complication rates for wound dehiscence and infection following open reduction and internal fixation are as high as 25%, with the highest rates observed among the elderly and patients with diabetes and vascular diseases [3–5]. These complications are often devastating for patients, requiring additional treatment and causing longer post-operative immobility. Several less-invasive techniques have been developed to reduce the risk of these complications, including intramedullary devices such as intramedullary screws and rush pins [6]. Biomechanical studies have shown that the intramedullary screw is as strong as plate osteosynthesis [2]. Furthermore, other studies have reported lower complication rates for intramedullary screws compared to standard plate osteosynthesis for patients with poor soft tissue conditions [7, 8]. However, few studies in the literature have evaluated the risk of complications associated with intramedullary nails and rush pins, and the aforementioned studies only included a limited number of patients. These limitations may explain—to some extent—why plate osteosynthesis is still the standard treatment for lateral malleolus fractures even in patients with fragile soft tissue.

The primary aim of this study was to estimate the risk of re-operation within three months of primary surgery among patients with unstable ankle fractures treated with fixation of the distal fibula with an intramedullary screw or rush pin. Our secondary aim was to estimate the risk of minor complications such as delayed bone union, infection, and delayed wound healing.

## Patients and methods

We conducted a retrospective, single-center study of an unselected historic cohort. We included patients who underwent minimally invasive surgery for an unstable ankle fracture and received either a 3.5-mm fully threaded intramedullary screw or a rush pin. Intramedullary screws and rush pins are primarily used as definitive surgery for patients with fragile soft tissue or high risk for secondary complications (e.g., the elderly and patients with diabetes mellitus or vascular disease) at our institution. We identified all patients above 18 years of age that were surgically treated for an ankle fracture at our institution from January 1, 2012, to December 31, 2018, by searching the hospital’s business intelligence system for the surgical procedure codes for any fracture management of the ankle (ICD-10 code KNHJ40–KNHJ88). These surgical procedure codes have been previously validated by our institution [9]. Data were extracted from the medical records during 2019 and 2020 allowing a minimum 2 years follow-up.

By review of the surgical procedure code and medical records we included all patients that were treated with either an intramedullary screw or rush pin in the distal fibula. We assessed patients’ medical records to exclude multi-traumatized patients, patients with distal tibia fractures (e.g., pilon fractures), and patients for whom the indication for using an intramedullary device was not due to vulnerable soft tissue or comorbidities (e.g. young patients with undisplaced fibula fractures). Demographic data were extracted from patients’ medical records, including information of whether patients were previously diagnosed with osteoporosis (Table 1).

**Table 1:** Demographic of study population (*N* = 80 patient)

*Operation Method*	*Screw*	*Rush pin*	*Total (N)*
	55	25	80
*Age*	73.3 (24–100)	79 (64–95)	75 (24–100)
*Sex*			
Male	12	9	21
Female	43	16	59
*AO*			
44-A2	6	1	7
44-A3	0	1	1
44-B1	2	0	2
44-B2	21	12	33
44-B3	23	10	33
44-C1	0	1	1
44-C2	1	0	1
Missing	2	0	2
*Lauge Hansen*			
SA2	5	2	7
SE2	2	1	3
SE3	3	0	3
SE4	43	20	63
PA3	0	1	1
PE3	1	1	2
Missing	1	0	1
*Comorbidities*			
0–1	26	13	39
2. – 4	26	10	36
>=5	3	2	5
*ASA-score*			
1	4	1	5
2	25	11	36
3	21	13	34
4	5	0	5
*Osteoporosis*			
Yes	41	19	60
No	14	6	20
*Trauma mechanism*			
Car Accident	1	1	2
Pedestrian/Cyclist	4	2	6
Fall on same level	48	22	70
Fall above 2 m	1	0	1
Missing	1	0	1

Perioperative radiographs for all identified patients were assessed, by the primary author, who is a consultant in orthopedic surgery, to confirm that patients were treated with either an intramedullary screw or a rush pin and to exclude patients that did not have ankle fractures, e.g., patients with distal tibia fractures. All medical records data were recorded in REDCap®.

**Fig. 1:**
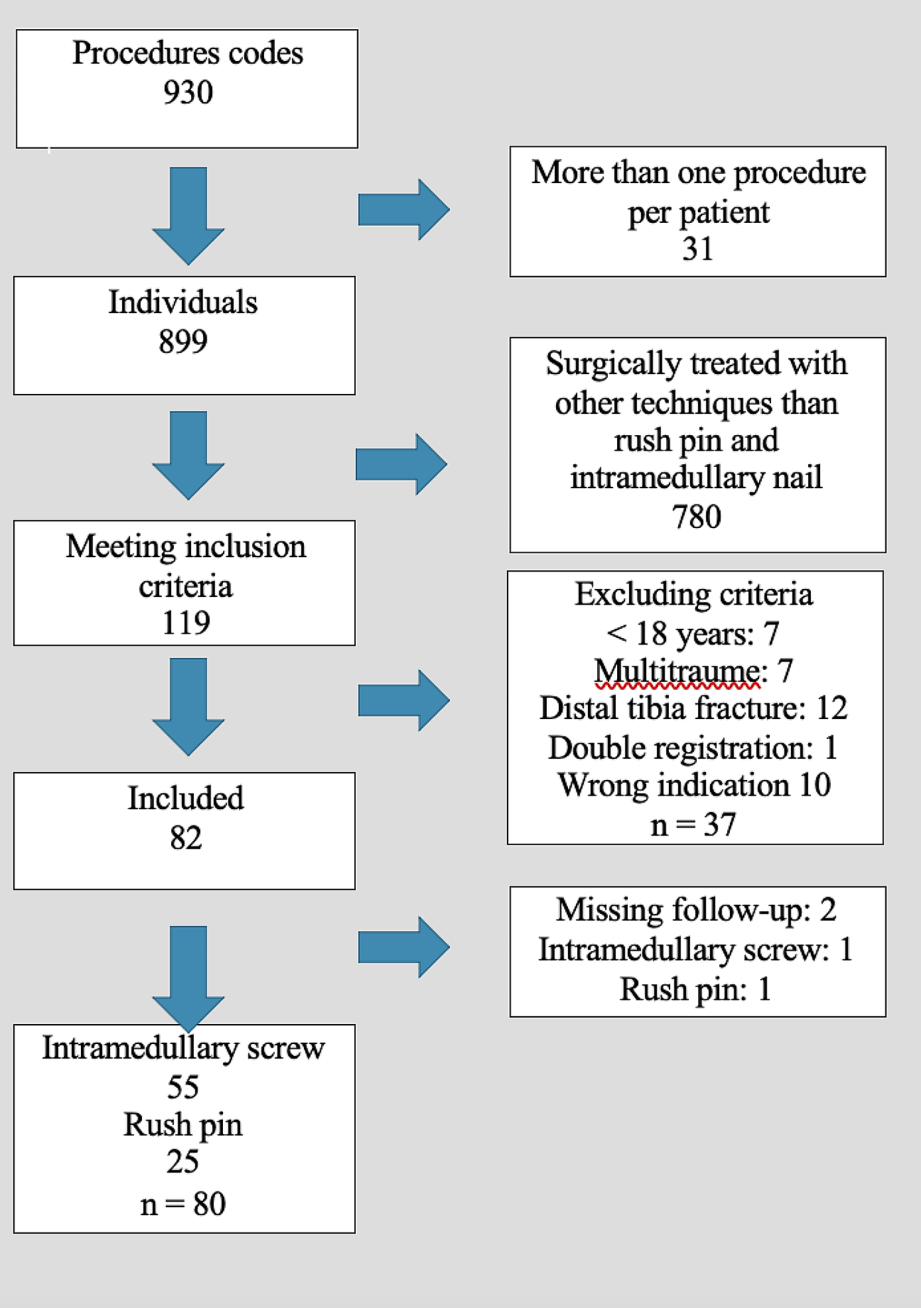
Flowchart of inclusion and exclusion criteria

A total of 930 ankle fracture surgeries across 899 individual patients were identified (KNHJ40–KNHJ88). Following a review of patients’ medical records and radiographs, 780 patients were excluded because they were treated with surgical techniques other than an intramedullary screw or a rush pin (see flowchart; (Fig. 1)). An additional 37 patients were excluded according to the aforementioned exclusion criteria, leaving 82 ankles of which two were lost to follow-up; these patients did not present at the outpatient clinic six weeks following surgery. Thus, 80 ankle fractures remained for evaluation in this study.

The primary outcome was a re-operation with hardware removal or re-osteosyntheses within the first three months following surgery. The indication for re-operation was recorded. The secondary outcome was a minor complication, defined as a delayed union (no visible union on radiographs acquired six weeks after surgery and extended immobilization), wound infection, or delayed wound healing (i.e., a wound did not close within the first six weeks following surgery).

### Operative technique

**Fig. 2:**
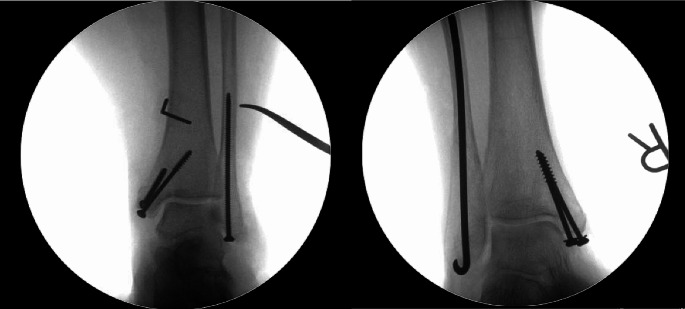
Left: Post-operative anterior-posterior view of a 3.5 mm intramedullary screw placed in the distal fibula. Right: Post-operative anterior-posterior view of a rush pin placed in the distal fibula

The patient is placed in the supine position. A small stab incision is made to access the distal part of the fibula. Using fluoroscopy as a guide, a soft tissue protector is placed on the distal part of the fibula and closed reduction of the lateral malleolus fracture is performed. The cortex is drilled with a 3-mm drill. A 3.5-mm fully threaded cortical screw is secured by hand, or a rush pin is inserted and secured with a hammer (Fig. 2). The wound is closed with 4 − 0 nylon sutures. All patients were put in a low leg cast or a walker boot and where not allowed weight bearing for at least 6 weeks. The procedure where preformed or supervised by a chief physician specialized in trauma orthopedics. Several different physicians performed the procedure. The decision to use a screw or a rush pin was made by the physician.

### Evaluating the stability of the reduction and osteosyntheses with radiographs six weeks post-operatively

**Fig. 3:**
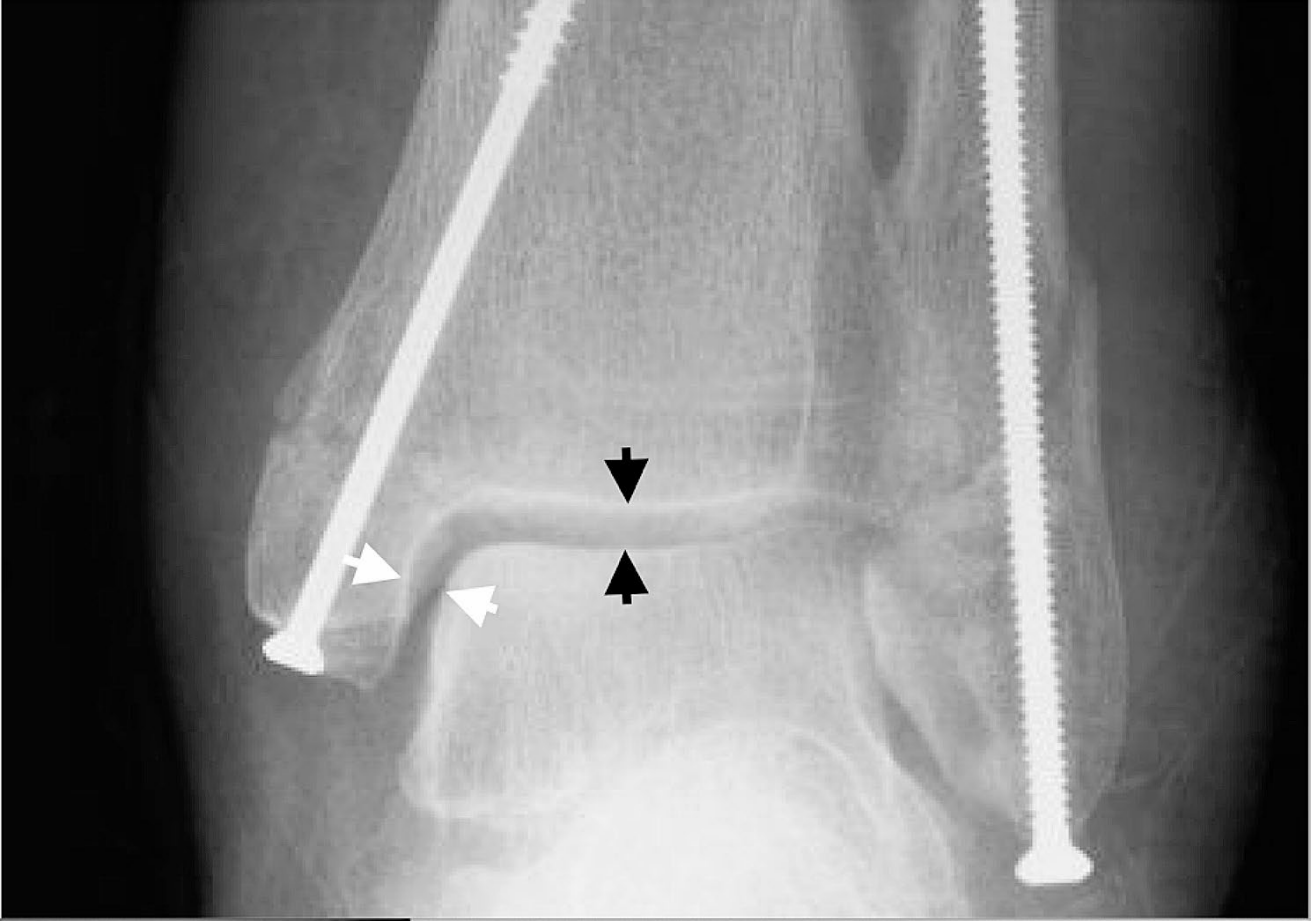
Anterior-posterior view of an ankle fracture with an intramedullary 3.5 mm screw. The medial clear space (white arrows) is < 5 mm. The medial clears space is smaller than the width of the superior clear space (black arrows)

The medial clear space was measured using anterior-posterior radiographs (Fig. 3). A medial clear space of more than 5 mm in total, or 1 mm wider than the superior clear space, was reported. We used these metrics because a medial clear space measuring more than 5 mm indicates instability in the ankle mortise [10–12].

The medial clear space was measured just below the medial corner of the talar dome to the closest edge of the lateral aspect of the medial malleolus. We measured the superior clear space in the middle of the talar dome where the concavity is lowest (Fig. 3). We also did a subjective evaluation regarding the reduction and considered it a loss of reduction if the authors would have offered a reoperation to a healthy patient. The measuring and evaluation was done by the primary author. Every measures and x-rays were then checked by the two co-authors, a trained orthopedic and a chief physician both specialized in trauma orthopedics.

## Results

Of the 80 patients included in this study, 55 (69%) were treated with a 3.5-mm intramedullary screw and 25 (31%) were treated with a rush pin. Most of the patients were female (59 patients) and the mean age was 75 (range 24 to 100) years. 41 patients had more than 2 comorbidities (cardiovascular disease, heart attack, stroke, diabetes, smoking, COPD, alcohol abuse, renal insufficiency, osteoporosis, high blood pressure, cancer and more).

The demographic characteristics of the patients are presented in Table 1.

Three patients (4%) underwent re-operation within three months due to either fracture displacement (two patients) or hardware cutout (one patient). One additional patient presented with fracture displacement as noted in the medical record but was treated conservatively due to considerable comorbidities. Furthermore, four patients (5%) had their hardware removed more than three months after their primary surgery, either due to deep infection (one patient) or hardware problems (three patients). Of the remaining 77 patients that did not undergo re-operation within three months, four (5%) had superficial wound infections and six (8%) experienced delayed wound healing.

During our evaluation of radiographs obtained at the outpatient clinic six weeks after surgery, we identified nine patients (11%) that experienced loss of reduction due to fracture displacement, despite not having undergone re-operation within three months of their primary surgery. A medial clear space larger than 5 mm was observed in 10 patients (13%), whereas 11 patients (14%) had a medial clear space that was more than 1 mm larger than the superior clear space (Table 2). The measured rush pin diameter ranges from 2.5 to 4.0 mm (mean 3.43 mm). Considering the concerningly high rate of malreduction, this technique may be best reserved for low demand elderly patients or those with severe complications. Patients should be appropriately counseled about the higher likelihood of malreduction and possible chronic ankle pain or worse functional outcome in long term.

**Table 2:** Reported complications and measured clear spaces (*N* = 78 due to missing radiographs on 2 patients)

*Reoperation*	Screw	Rush pin	Total
Fracture displacement	1	1	2
Hardware cutout	1	0	1
Amputation	0	0	0
Compartment Syndrome	0	0	0
Deep infection	0	0	0
*Minor/early complication*			
Wound infection	3	1	4
Delayed wound healing	3	3	6
*Evaluation of follow-up* radiographs			
Loss of reduction	9	3	12
Unchanged	44	22	66
Missing	2	0	2
*Medial clear space*			
>=5 mm	7	3	10
< 5 mm	45	21	66
Missing	3	1	4
*Clear space difference between medial and above talus*			
> 1 mm	8	3	11
<= 1 mm	44	21	65
Missing	3	1	4

## Discussion

In this study, we identified 80 patients who were treated with an intramedullary fully threaded screw or rush pin. Only three patients (4%) underwent re-operation within three months and an additional 10 patients (13%) experienced complications such as delayed wound healing or superficial infection. However, a review of the patients’ radiographs obtained at follow-up showed that an additional nine patients (11%) had loss of reduction despite not having undergone re-operation within three months.

We found a low three-month re-operation rate in this study. In addition to the three patients that underwent re-operation, four patients (5%) had their hardware removed more than three months after their primary surgery. Given that a percutaneous intramedullary surgical technique is primarily used in patients with fragile skin conditions, a re-operation rate of 4% should be considered satisfactory and the method regarded as safe. Indeed, the standard technique of open reduction and fixation with a plate and screws has a considerably higher risk of re-operation [13]. An additional advantage of intramedullary screws and rush pins is that it is possible to perform surgery even when the leg is swollen. Thus, the time from trauma to surgery is reduced, in turn reducing the total duration of a patient’s hospital stay and lowering costs. Furthermore, the procedure is less invasive to the soft tissue.

The re-operation rate we observed for patients treated with intramedullary screws or rush pins is comparable to that reported for patients treated with fibular nails [14–16]. This result was expected because the surgical methods are similar. As the cost of fibular nails is much higher than the screws and rush pins, the latter method might be a more economical option. An advantage of fibular nails over intramedullary screws and rush pins is that they enable fixation of the syndesmosis. This feature may strengthen the stability of the fixation, but studies directly comparing the two methods are lacking. At our institution, we have not used intramedullary screws or rush pins in combination with syndesmotic screws as it against our guidelines. However, it has come to our attention that other departments combine intramedullary screws or rush pins with a syndesmotic screw and this might be a valuable solution.

The re-operation rate of 4% found in this study are comparable to the rate reported by Smith et al. from 2017 [17], who found a re-operation rate of 4%, and to the rate reported in a systematic review by Loukachov et al. in 2017 [7], which included six studies with a total of 180 patients. In this review, a loss of reduction was seen in two patients (1.1%), implant removal was necessary for three patients (1.7%), non-union was seen in two patients (1.1%), and only one patient (0.6%) presented with a wound infection. Loukachoy et al. [7] describe only loss of reduction in 1,1% compared to our finding of 15%. In Loukachoy study they used either a 3.5 or 4.5 mm screw. The 4.5 screw might be a better solution in older women with a wider intramedullary canal and this could explain the lower risk of loss of reduction they found in their study.

Subsequently, Ebraheim et al. [8] retrospectively reviewed data from 45 patients with distal fibular fractures who were treated with a cannulated intramedullary screw. The average follow-up time was six months. Two patients required secondary surgery, one due to non-union and the other due to pain from the screws.

A recent prospective study by Zawam et al. [18] comparing an percutaneous intramedullary screw to the traditional neutralizations plate reported no significant difference in functional outcome between the two groups.

Albana et al. [19] Retrospectively reviewed data from 37 patients treated with a 150 mm treated intramedullary screw with similar risk factors as reported in this study. Albana et al. report union in 97.3% and total complications observed in 15.8%. Albane et al. conclude, that an intramedullary screw is a good alternative to plate and screws in patients with known risk factors for wound complications.

Despite the observed low risk of re-operation, the relatively high percentage of loss of reduction (12/80 patients; 15%) is concerning. The fact that only three patients underwent re-operation, but that an additional nine patients showed loss of reduction on their follow-up radiographs, may be due to the fragile patient category. This observation underscores that it is important to not focus exclusively on re-operation rates when performing studies of surgical methods used in fragile patients.

This retrospective study has some limitations, including a lack of a randomly selected control group. A second limitation is that the data are limited to patients’ radiographs and medical records. Thus, although the probability of missing data pertaining to patients’ three-month re-operation status and radiographically evaluated stability is very low, there is a moderate risk that the prevalence of delayed wound healing and infections is underestimated as these events are more likely to be insufficiently described in medical records. A third limitation to this study is that there probably is a high rate of undiagnosed osteoporosis in this group of patients, which could have a negative effect on the rate a loss of reduction. Furthermore, two patients were lost to follow-up in this study. However, we believe that the risk of losing the remaining 80 patients to follow-up is quite small because their unique Danish civil registration numbers allow us to identify all contacts within the Central Jutland region. Therefore, we know that they did not undergo re-operation within this region, nor did they present at another hospital with minor complications.

A major limit for this study is the lack of patient reported outcome measures (PROM) and functional outcome at follow-up. Unfortunately, these information’s was not well described or defined in the medical record to report in the study. A prospective comparative study reporting PROM’s and functional outcome are needed.

In conclusion, we found that intramedullary fixation of distal fibula fractures with either screws or rush pins exhibits low re-operation rates. However, there is a concerningly high proportion of patients that presented with radiological loss of reduction. Additional studies, including randomized controlled trials to compare the different minimally invasive intramedullary fixation methods for distal fibula fractures among patients with fragile skin conditions, represent important directions for future research.

## Brief summery

- What Is Already Known.
  - Ankle fractures are a common acute fracture.
  - Fragile patients with comorbidities and ankle fractures have high complication rates when treated with open reduction and internal fixation.
  - Intramedullary fixation of the distal fibula has proven to be mechanically stable.
- What This Study Adds.
  - Only 4% was reoperated within 3 months.
  - Low complication rates regarding wound healing and skin infections.
  - A high proportion of patients presented with radiological loss of reduction after 6 weeks.

### Electronic supplementary material

Below is the link to the electronic supplementary material.

Supplementary Material 1

Supplementary Material 2

Supplementary Material 3

Supplementary Material 4

## References

[1] Elsoe R, Ostgaard SE, Larsen P (2018) Population-based epidemiology of 9767 ankle fractures. Foot Ankle Surg. 24(1) 10.1016/j.fas.2016.11.002 29413771

[2] Bankston AB, Anderson LD, Nimityongskul P (1994) Intramedullary Screw fixation of lateral malleolus fractures. Foot Ankle Int. 15(11) 10.1177/107110079401501105 7849975

[3] White TO, Bugler KE, Appleton P, Will E, McQueen MM, Court-Brown CM (2016) A prospective randomised controlled trial of the fibular nail versus standard open reduction and internal fixation for fixation of ankle fractures in elderly patients. Bone Joint J 98-B(9). 10.1302/0301-620X.98B9.35837 27587528

[4] SooHoo NF, Krenek L, Eagan MJ, Gurbani B, Ko CY, Zingmond DS (2009) Complication rates following open reduction and internal fixation of ankle fractures. J Bone Joint Surg. 91(5) 10.2106/JBJS.H.00653 19411451

[5] Zaghloul A, Haddad B, Barksfield R, Davis B (2014) Early complications of surgery in operative treatment of ankle fractures in those over 60: a review of 186 cases. Injury. 45(4) 10.1016/j.injury.2013.11.008 24388418

[6] Pritchett JW (1993) Rush rods versus plate osteosyntheses for unstable ankle fractures in the elderly. Orthop Rev. 22(6) 8351172

[7] Loukachov Vv, Birnie MFN, Dingemans SA, de Jong VM, Schepers T (2017) Percutaneous Intramedullary Screw fixation of distal fibula fractures: a Case Series and systematic review. 56, J Foot Ankle Surg 10.1053/j.jfas.2017.05.024 28647520

[8] Ebraheim NA, vander Maten JW, Delaney JR, White E, Hanna M, Liu J (2019) Cannulated Intramedullary Screw fixation of distal fibular fractures. Foot Ankle Spec. 12(3) 10.1177/1938640018790082 30091366

[9] Gundtoft PH, Danielsson FB, Houlind M, Mortensen SO, Corap Y, Bonde N (2022). The positive predictive value of operative treated ankle fracture diagnoses and Surgical Procedure codes in the Danish National Patient Registry. Dan Med J.

[10] Park SS, Kubiak EN, Egol KA, Kummer F, Koval KJ (2006) Stress radiographs after ankle fracture: the effect of ankle position and deltoid ligament status on medial clear space measurements. J Orthop Trauma. 20(1) 10.1097/01.bot.0000189591.40267.09 16424804

[11] Schock HJ, Pinzur M, Manion L, Stover M (2007) The use of gravity or manual-stress radiographs in the assessment of supination-external rotation fractures of the ankle. J Bone Joint Surg - Ser B. 89(8) 10.1302/0301-620X.89B8.19134 17785745

[12] Weber M, Burmeister H, Flueckiger G, Krause FG (2010) The use of weightbearing radiographs to assess the stability of supination-external rotation fractures of the ankle. Arch Orthop Trauma Surg. 130(5) 10.1007/s00402-010-1051-1 20082083

[13] Jacobsen GH, Gude MH, Viberg B, Gundtoft PH (2022) Risk of reoperation in simple ankle fracture surgery when comparing locking plate with nonlocking plate. J Foot Ankle Surg. 61(3) 10.1053/j.jfas.2021.10.008 34838457

[14] Asloum Y, Bedin B, Roger T, Charissoux JL, Arnaud JP, Mabit C (2014) Internal fixation of the fibula in ankle fractures. A prospective, randomized and comparative study: Plating versus nailing. Orthopaedics and Traumatology: Surgery and Research. 100(4S) 10.1016/j.otsr.2014.03.005 24709304

[15] Carter TH, Wallace R, Mackenzie SA, Oliver WM, Duckworth AD, White TO (2020) The fibular intramedullary nail Versus Locking plate and lag screw fixation in the management of unstable Elderly Ankle fractures: a cadaveric biomechanical comparison. J Orthop Trauma. 34(11) 10.1097/BOT.0000000000001814 33065664

[16] Smith G, Mackenzie SP, Wallace RJ, Carter T, White TO (2017) Biomechanical comparison of Intramedullary Fibular nail Versus plate and screw fixation. Foot Ankle Int. 38(12) 10.1177/1071100717731757 28971694

[17] Smith M, Medlock G, Johnstone AJ (2017) Percutaneous screw fixation of unstable ankle fractures in patients with poor soft tissues and significant co-morbidities. Foot Ankle Surg. 23(1) 10.1016/j.fas.2015.11.008 28159037

[18] Zawam SH, Mabrouk MG, El-Desouky MA (2022). Lateral malleolar fractures Weber Type A and B: does percutaneous intramedullary screw confer a solid alternative to the traditional neutralization plate?. Int Orthop.

[19] Albana MF, Jimenez ML, Brill BJ, Principe MJ, Quercetti NF (2022). Indications for retrograde intramedullary screw fixation of the distal fibula: a retrospective cohort series. OTA Int.

