# Supplementary material for: Percutaneous intramedullary screw or rush pin fixation of unstable ankle fractures in patients with fragile soft tissue – retrospective study of 80 cases
Source: Arch Orthop Trauma Surg. 2024 Apr 13;144(5):2157–63. doi: 10.1007/s00402-024-05290-w (PMC11093783; doi:10.1007/s00402-024-05290-w)
Supplement: Supplementary file 1 — Supplementary Material 1 [file 402_2024_5290_MOESM1_ESM.pdf]

EDITORIAL CERTIFICATE

Authors: Per Gundtoft

Document title: Percutaneous intramedullary screw or rush pin fixation of unstable ankle fractures in fragile people – retrospective study of 80 cases

Date Issued: 3 Nov 2022

Cambridge Proofreading LLC

This document certifies that the above manuscript was proofread and edited by Cambridge Proofreading LLC.

This document certifies that the above manuscript was proofread and edited by Cambridge Proofreading Worldwide LLC. The document was edited for proper English language, grammar, punctuation, spelling, and overall style by one or more of our academic editors. The editor endeavoured to ensure that the author's intended meaning was not altered during the review. All amendments were tracked with the Microsoft Word 'Track Changes' feature. Therefore, the authors had the option to reject or accept each change individually.

Kind regards,
Cambridge Proofreading

Cambridge Proofreading Worldwide LLC is a registered company headquartered in Chicago, Illinois, USA with a global presence. All of our editors are native speakers from USA and the UK. Our Certificate of Good Standing can be found in the Illnois state business database by searching our name here.
